# Supplementary material for: Acid-Triggered Switchable Near-Infrared/Shortwave Infrared Absorption and Emission of Indolizine-BODIPY Dyes
Source: Molecules. 2023 Jan 29;28(3):1287. doi: 10.3390/molecules28031287 (PMC9919721; doi:10.3390/molecules28031287)
Supplement: Supplementary file 1 [file molecules-28-01287-s001.zip › molecules-2063161-supplementary.pdf]

Supporting Information

Acid-Triggered Switchable Near-Infrared/Shortwave Infrared Absorption and Emission of Indolizine-BODIPY Dyes

Matthew A. Saucier,[1] Cameron Smith,[1] Nicholas A. Kruse,[1] Nathan I. Hammer,[1] and Jared H. Delcamp[1,2,3]*

1 Department of Chemistry and Biochemistry, University of Mississippi, University, Mississippi 38677
2 Current Address: Materials and Manufacturing Directorate, Air Force Research Laboratory, 2230 Tenth Street Area B Building 655, Wright-Patterson AFB, OH 45433, USA
3 Current Address: UES, Inc., 4401 Dayton Xenia Rd, Dayton, OH 45432, USA

* Correspondence: delcamp@olemiss.edu; jared.delcamp.ctr@us.af.mil

Table of Contents

NMR spectra for new compounds 3

Figure S1. ^{1}H-NMR (400 MHz, $CDCl_3$) of **1Ph-CHO**. 3

Figure S2. ^{13}C-NMR (101 MHz, $CDCl_3$) of **1Ph-CHO**. 3

Figure S3. ESI-HRMS spectrum of **1Ph-CHO**...4

Figure S4. ^{1}H-NMR (300 MHz, DMSO-d_6) of **2Ph**. 5

Figure S5. ^{19}F-NMR (376 MHz, DMSO-d_6) of **2Ph**. 5

Figure S6. ^{11}B NMR (128 MHz, DMSO-d_6) of **2Ph**. 6

Figure S7. ESI-HRMS spectrum of **2Ph**...6

Figure S8. ^{1}H-NMR (400 MHz, DMSO-d_6) of **1Ph**...7

Figure S9. ^{19}F-NMR (376 MHz, DMSO-d_6) of **1Ph**. 7

Figure S10. ^{11}B-NMR (128 MHz, DMSO-d_6) of **1Ph**...8

Figure S11. ESI-HRMS spectrum of **1Ph**...9

Computational studies of BODIPY dyes 10

Table S1. Tabulated computational data **10**

Photophysical studies of BODIPY dyes...11

Figure S12. Solution stability studies 11

Figure S13. Absorption spectra of **DMA-TFA** over 4 d...11

Figure S14. Absorption spectra of **2Ph-TFA** over 4 d 12

Figure S15. Absorption spectra of **1Ph-TFA** over 4 d 12

Figure S16. Absorption of **DMA**, **DMA-TFA**, and **DMA-TFA-TEA**...13

Figure S17. Absorption of **2Ph**, **2Ph-TFA**, and **2Ph-TFA-TEA**...13

Figure S18. Absorption spectra of **2Ph**, **2Ph-TFA**, and **2Ph-AcOH** 14

Figure S19. Absorption spectra of **1Ph**, **1Ph-TFA**, and **1Ph-AcOH** 14

Figure S20. Full spectrum UV-Vis-NIR absorption and emission for **DMA**, **2Ph**, and **1Ph**.........15

NMR spectra for new compounds

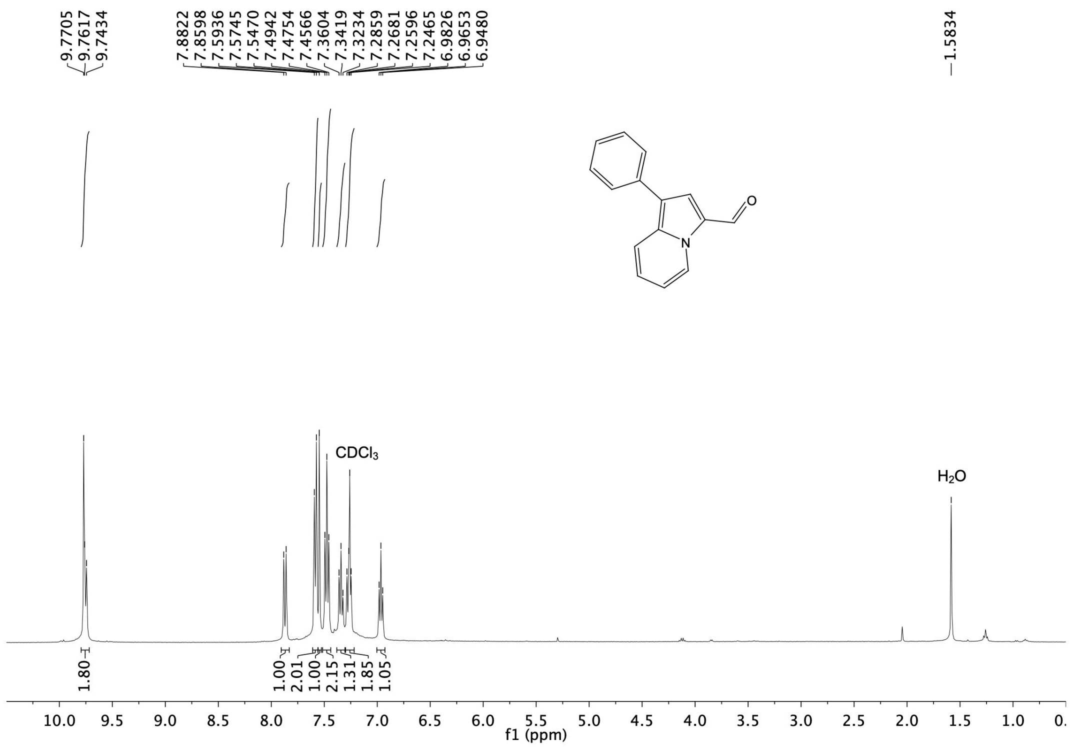

Figure S1. ^{1}H-NMR (400 MHz, $CDCl_3$) of **1Ph-CHO**.

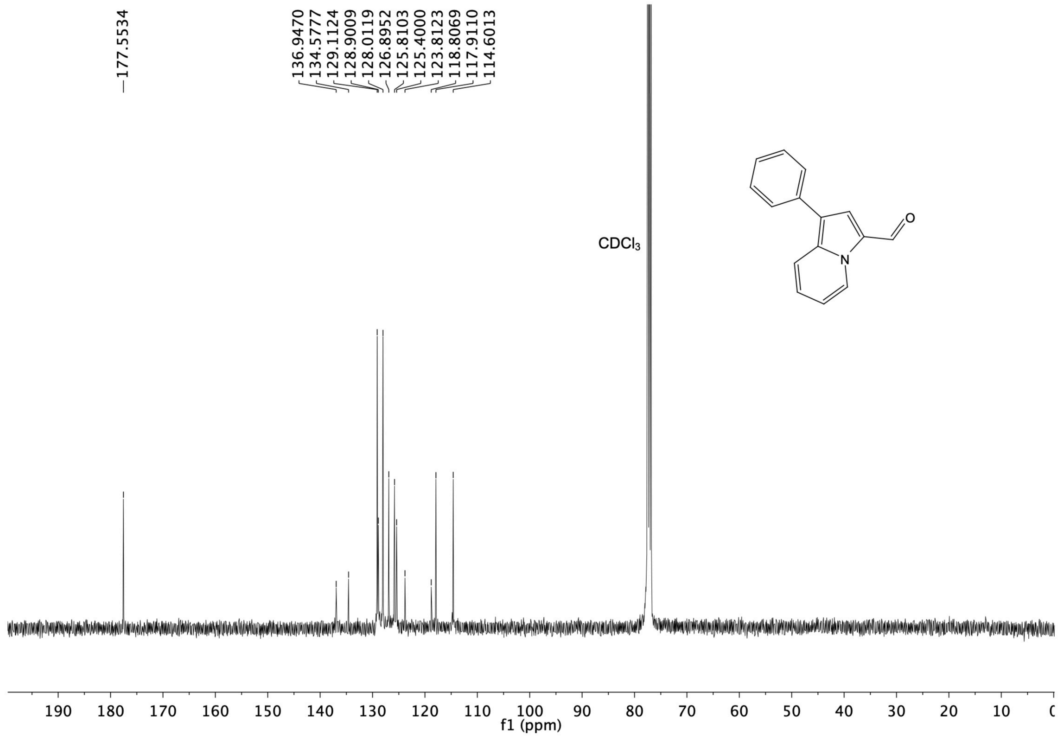

Figure S2. ^{13}C-NMR (101 MHz, $CDCl_3$) of **1Ph-CHO**.

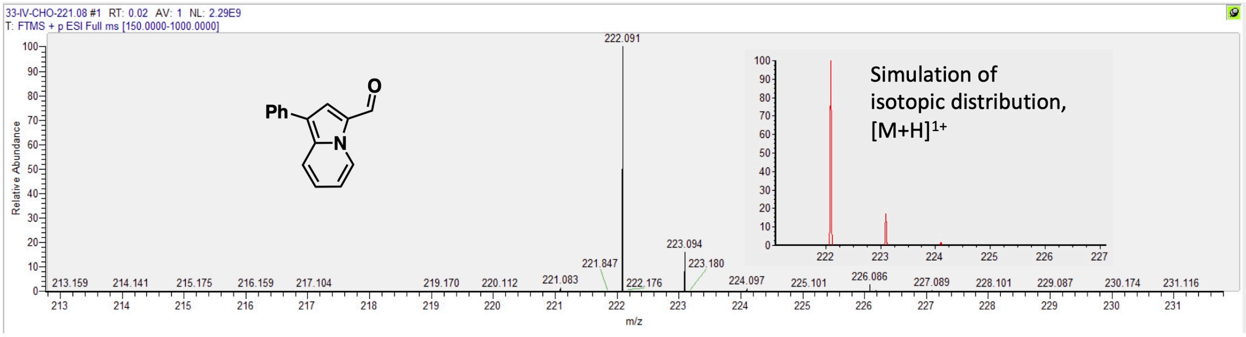

Figure S3. ESI-HRMS spectrum of **1Ph-CHO**, with an insert of the simulated spectrum for $[M+H]^{1+}$.

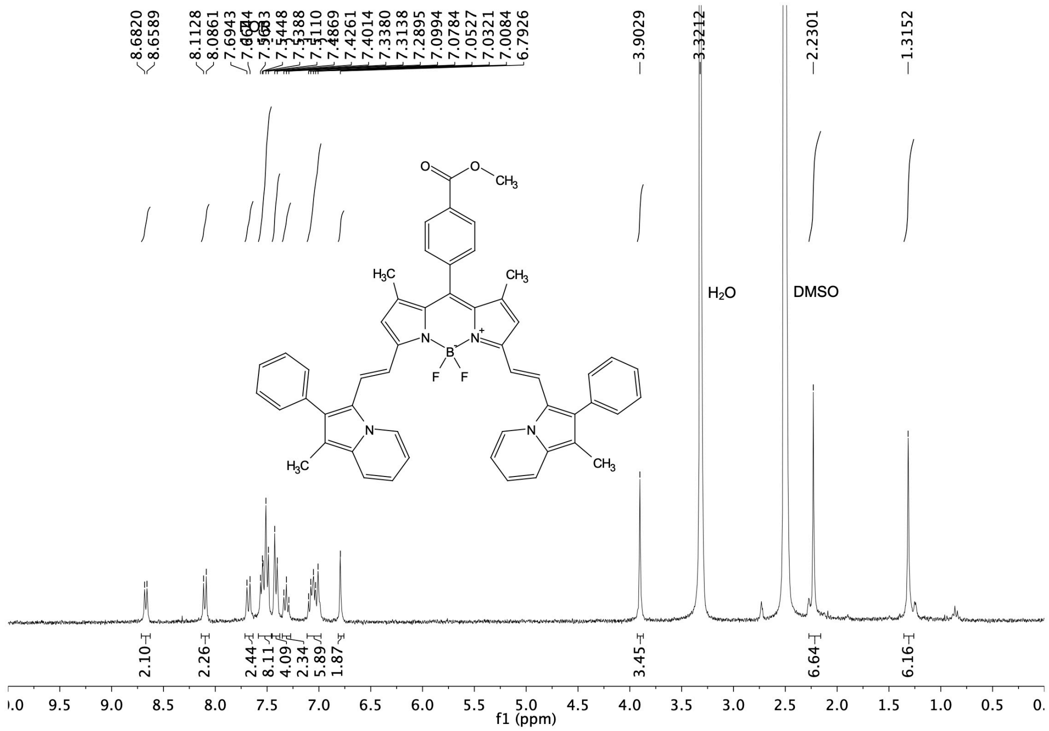

Figure S4. ^{1}H-NMR (300 MHz, DMSO-d_6) of **2Ph**.

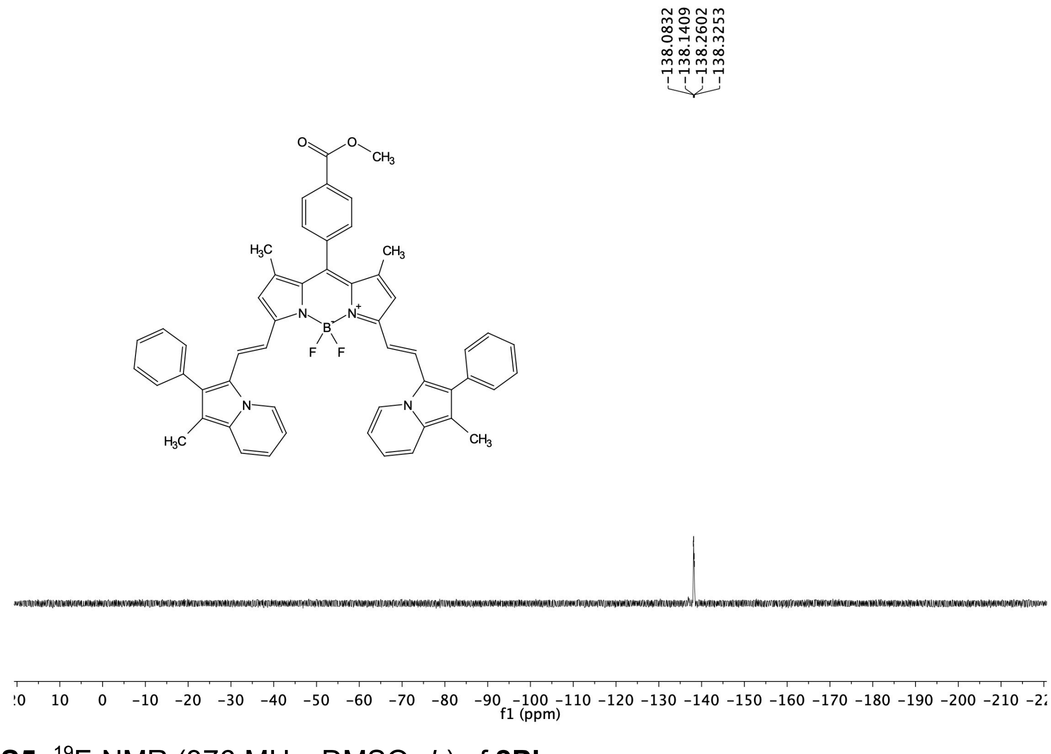

Figure S5. ^{19}F-NMR (376 MHz, DMSO-d_6) of **2Ph**.

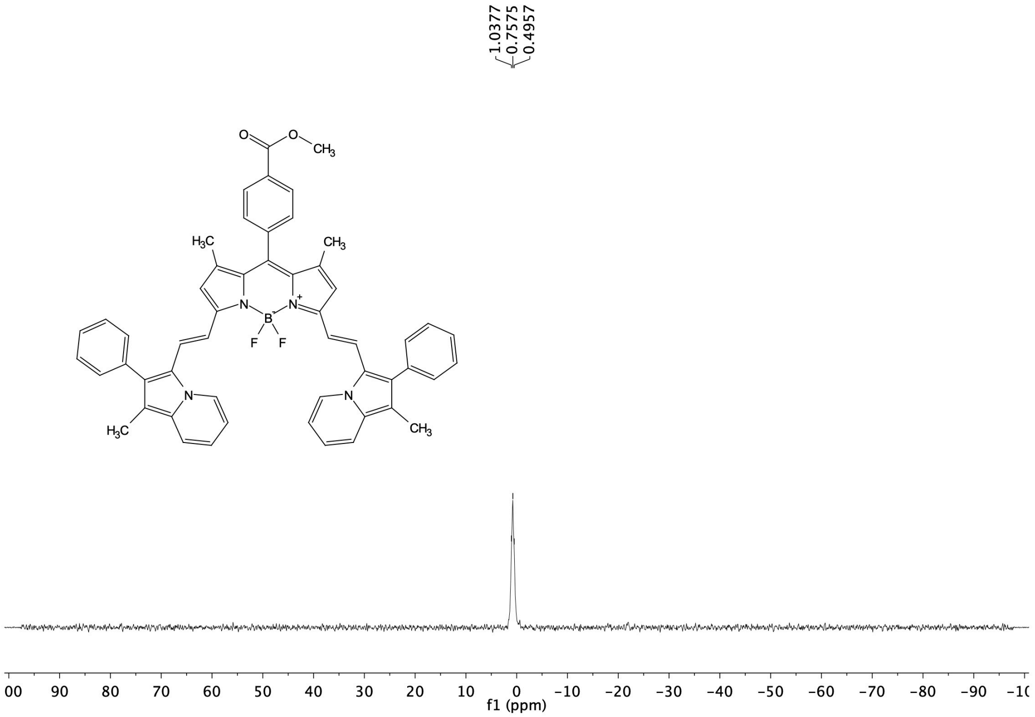

Figure S6. ^{11}B NMR (128 MHz, DMSO-d_6) of **2Ph**, recorded in a quartz NMR tube.

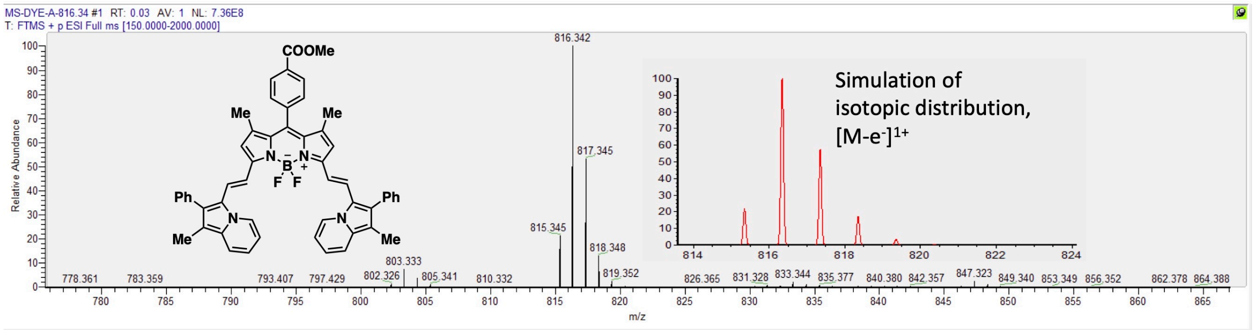

Figure S7. ESI-HRMS spectrum of **2Ph**, with simulated spectrum for $[M\text{-}e]^{1+}$ in inset.

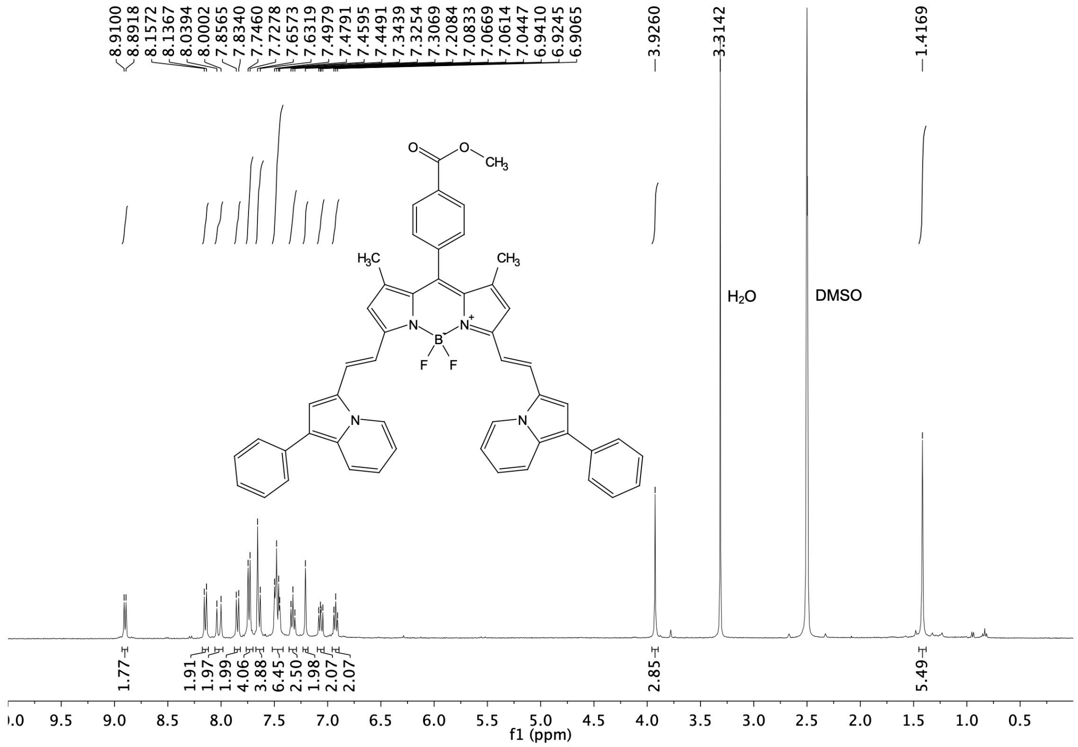

Figure S8. ^{1}H-NMR (400 MHz, DMSO-d_6) of **1Ph**.

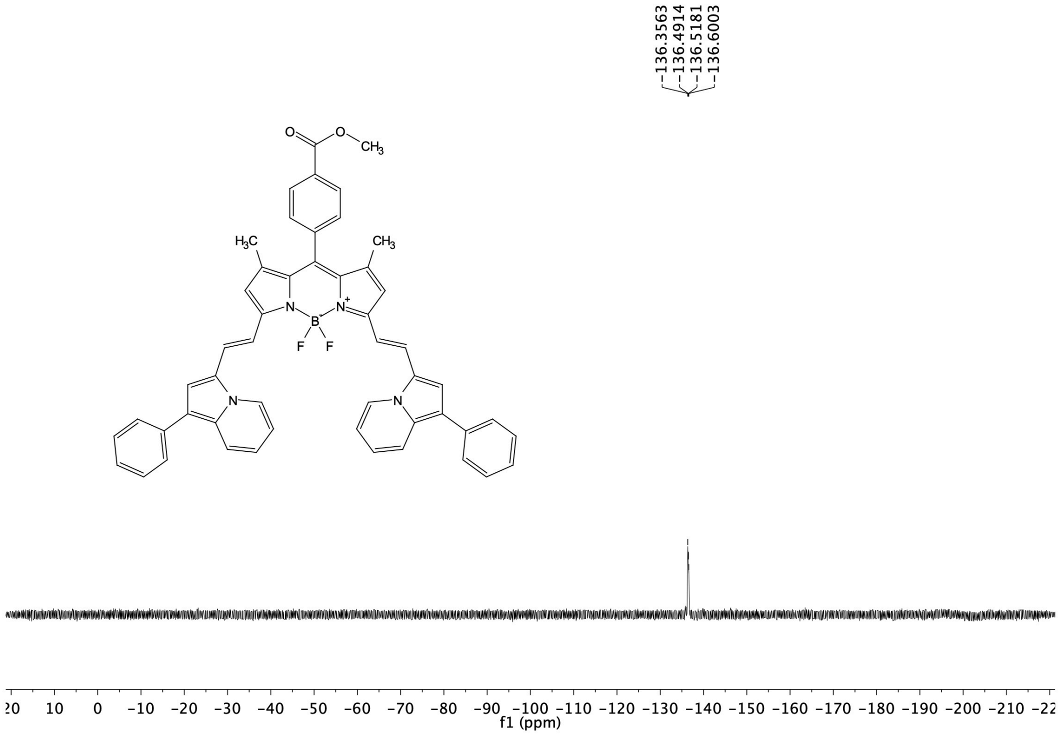

Figure S9. ^{19}F-NMR (376 MHz, DMSO-d_6) of **1Ph**.

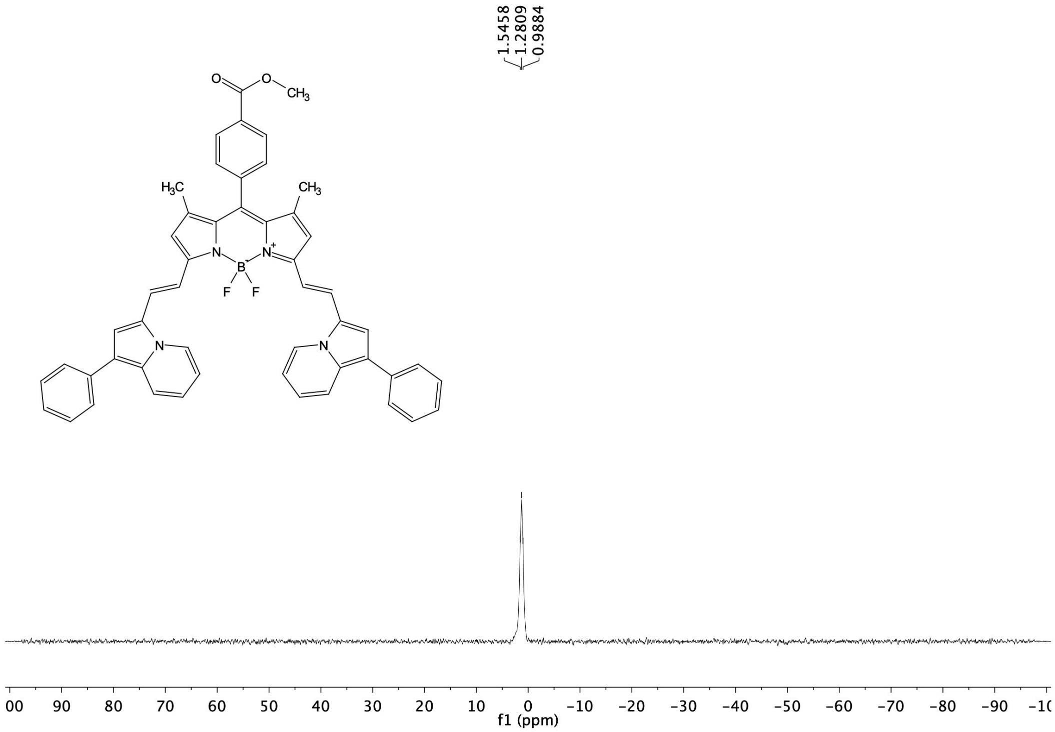

Figure S10. ^{11}B-NMR (128 MHz, DMSO-d_6) of **1Ph**, recorded in a quartz NMR tube.

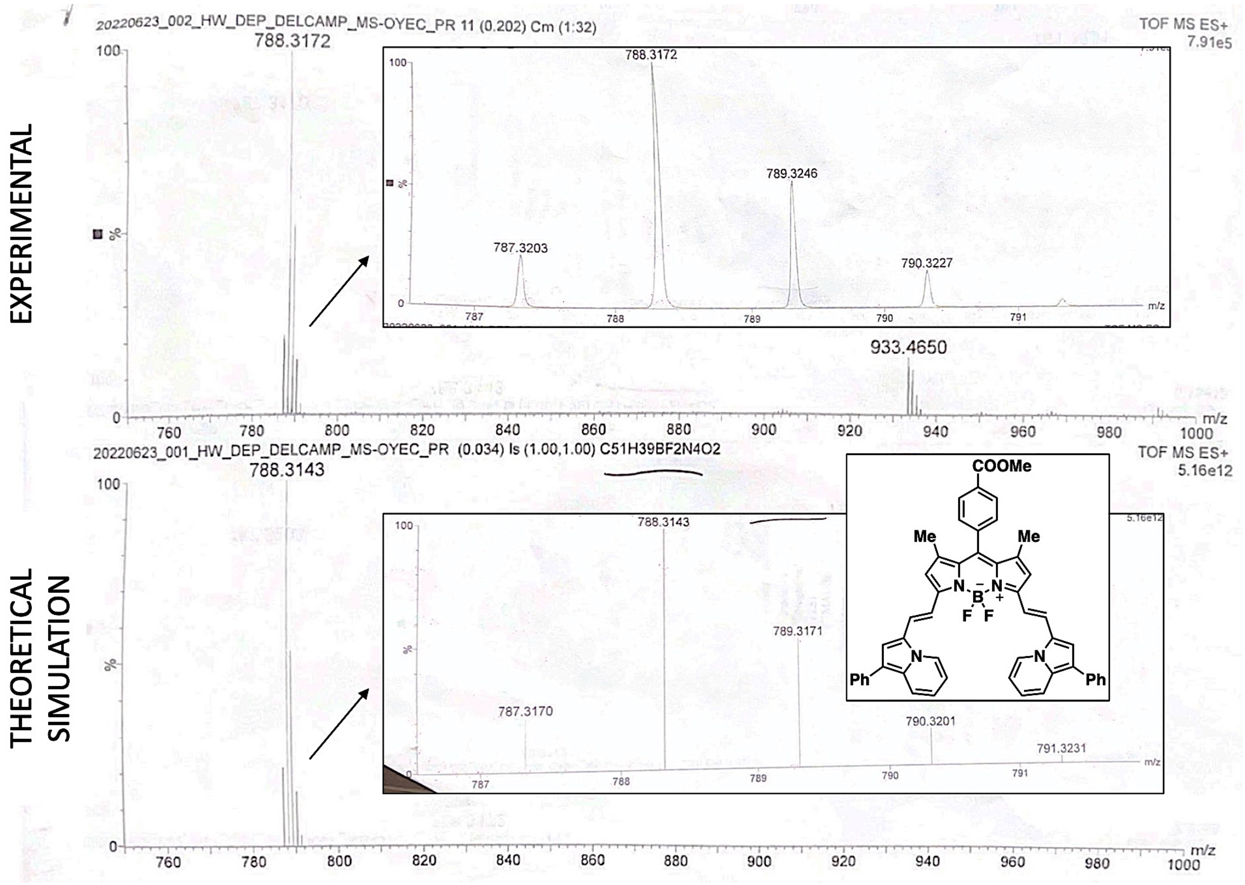

Figure S11. ESI-HRMS spectrum of **1Ph**, with simulated spectrum for $[M-e]^{1+}$ below the experimental spectrum. The M ion peaks are enlarged in the inset.

Computational studies of BODIPY dyes

Table S1. Tabulated computational data from TD-DFT of optimized geometries at the B3LYP/6-311G(d,p) level of theory with dichloromethane implicit solvation. Data from the lowest energy transition (i.e. HOMO to LUMO).

Dye	HOMO (eV)	LUMO (eV)	Transition	Contribution	Vertical Transition (nm | eV)	Oscillator Strength
**DMA**	-4.73	-2.78	$S_0 \rightarrow S_1$	100%	708 | 1.75	1.0969
**2Ph**	-4.68	-2.82	$S_0 \rightarrow S_1$	100%	765 | 1.62	0.8856
**1Ph**	-4.77	-2.89	$S_0 \rightarrow S_1$	100%	761 | 1.63	0.8591
**1Ph-TFA**	-5.51	-4.02	$S_0 \rightarrow S_1$	101%	954 | 1.30	0.5789

Photophysical studies of BODIPY dyes

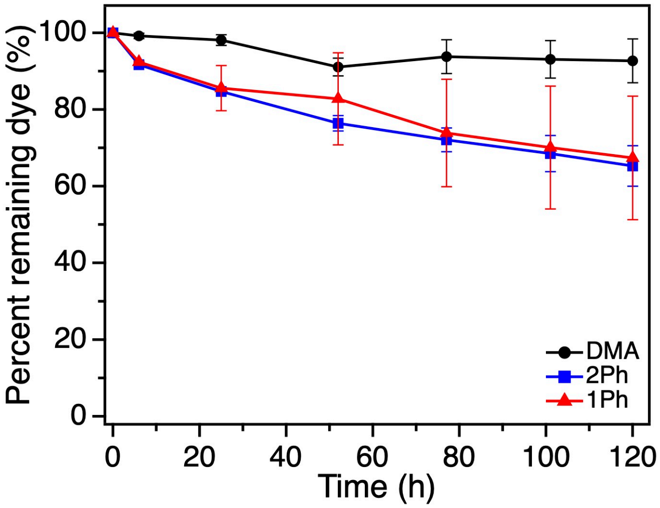

Figure S12. Solution stability studies. Percent of original absorption value of λ_{max}^{abs} for the three BODIPY dyes (**DMA** = 710 nm, **2Ph** = 801 nm, **1Ph** = 798 nm) in a sealed tube with DCE, in the dark, under ambient atmosphere, and at room temperature. Each experiment was run in duplicate with error bars reflecting the standard deviation from the average.

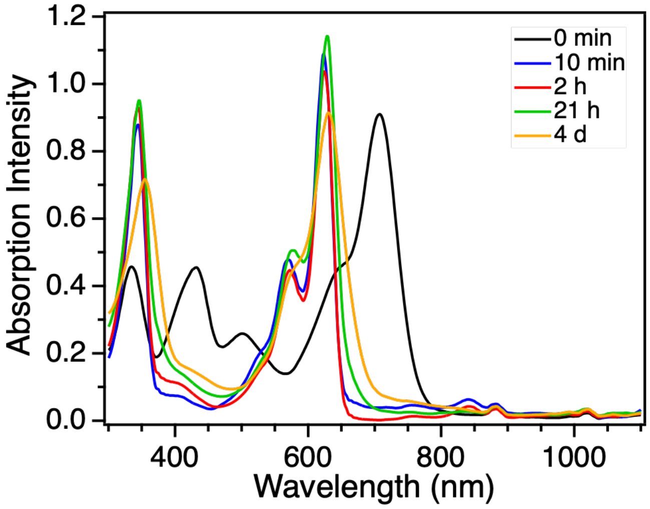

Figure S13. Absorption spectra of **DMA-TFA** over 4 d (ca. 10 μM dye in CH_2Cl_2 with 1% TFA [v/v]).

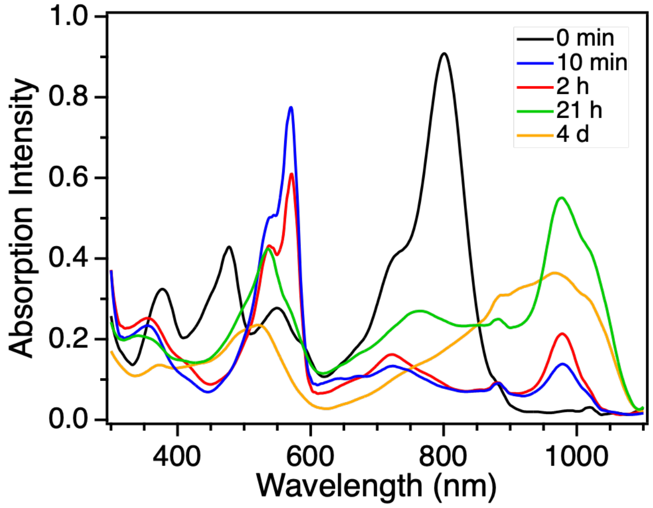

Figure S14. Absorption spectra of **2Ph-TFA** over 4 d (ca. 10 μM dye in CH_2Cl_2 with 1% TFA [v/v]).

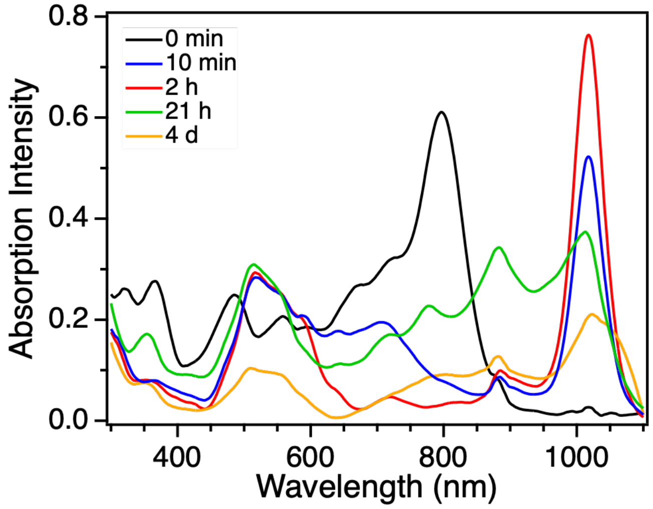

Figure S15. Absorption spectra of **1Ph-TFA** over 4 d (ca. 10 μM dye in CH_2Cl_2 with 1% TFA [v/v]).

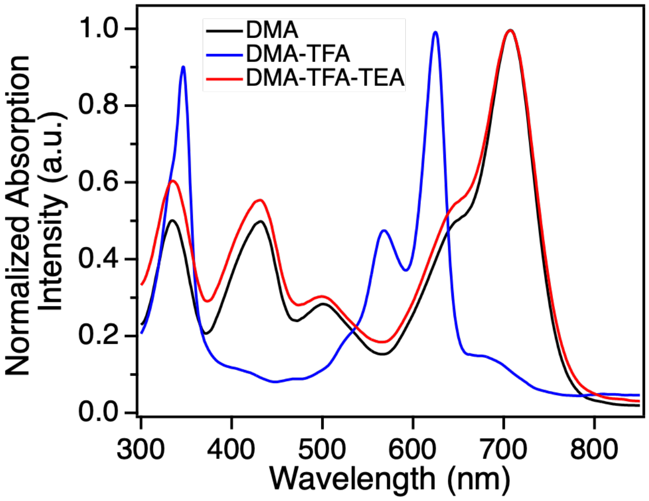

Figure S16. Absorption of **DMA**, **DMA-TFA**, and **DMA-TFA-TEA**. **DMA** was dissolved in DMSO-d_6 solution (ca. 3 mM), then 4,000 equiv. TFA was added and mixed thoroughly, then 4,000 equiv. TEA was added and mixed thoroughly. A small sample of each solution was taken and diluted with CH_2Cl_2 to produce the absorption spectrum.

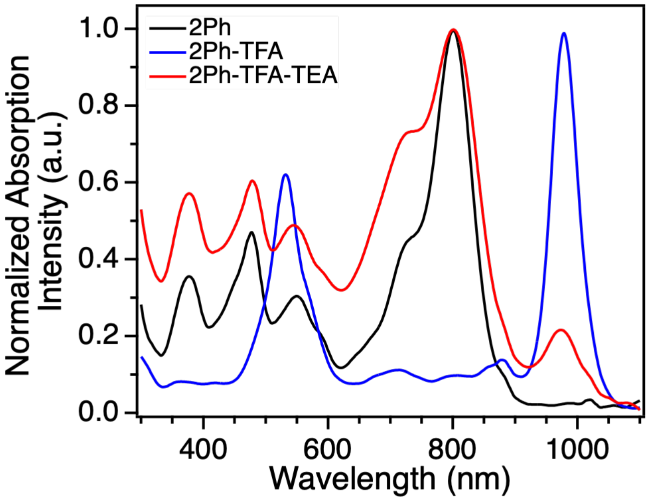

Figure S17. Absorption of **2Ph**, **2Ph-TFA**, and **2Ph-TFA-TEA**. **2Ph** was dissolved in DMSO-d_6 solution (ca. 3 mM), then 4,000 equiv. TFA was added and mixed thoroughly, then 4,000 equiv. TEA was added and mixed thoroughly. A small sample of each solution was taken and diluted with CH_2Cl_2 to produce the absorption spectrum.

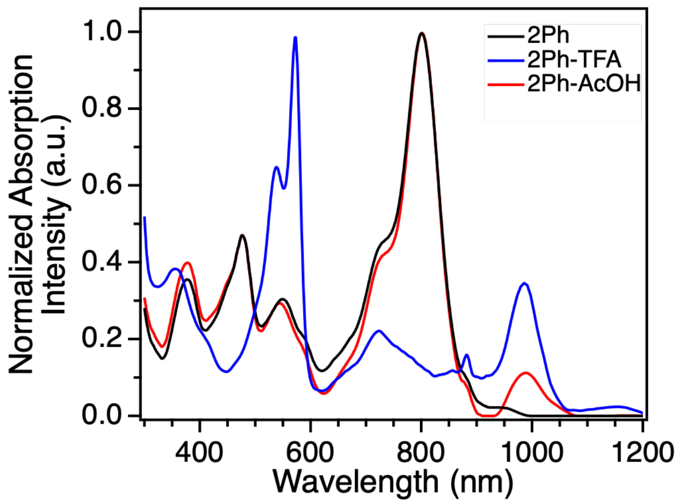

Figure S18. Absorption spectra of **2Ph** (CH_2Cl_2), **2Ph-TFA** (CH_2Cl_2 with 1% TFA [v/v]), and **2Ph-AcOH** (CH_2Cl_2 with 1% AcOH [v/v]) all after 2 h.

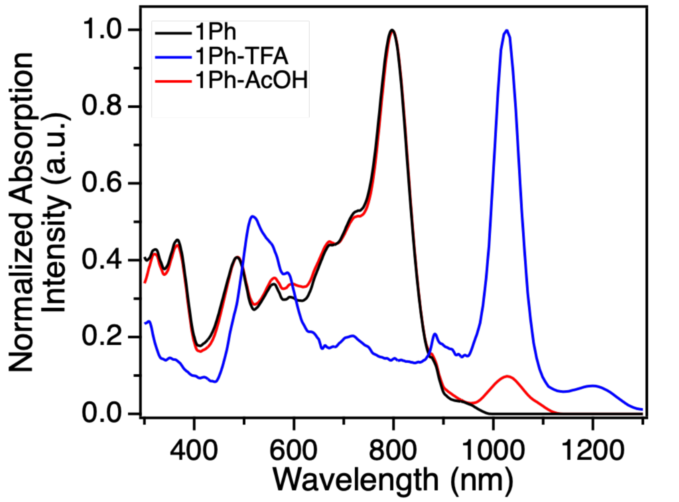

Figure S19. Absorption spectra of **1Ph** (CH_2Cl_2), **1Ph-TFA** (CH_2Cl_2 with 1% TFA [v/v]), and **1Ph-AcOH** (CH_2Cl_2 with 1% AcOH [v/v]) all after 2 h.

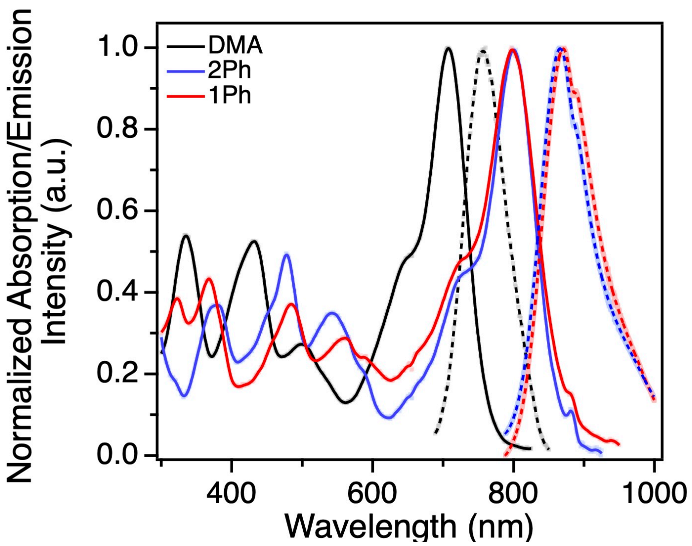

Figure S20. Full spectrum UV-Vis-NIR absorption and emission curves for the three BODIPY dyes (**DMA**, **2Ph**, and **1Ph**) in CH_2Cl_2.
